# The National Association of Railway Surgeons

**Published:** 1893-07

**Authors:** 


THE NATIONAL ASSOCIATION OF RAILWAY SURGEONS.—This Association met May 31st and June 1st and 2d, at Omaha, Nebraska. At the third day's session, after lively discussion, a resolution was passed favoring National, rather than State, control of quarantine. The following officers were elected for next year: President, Dr. W. J. Galbraith; First Vice-President, Dr. E. R. Lewis; Second Vice-President, Dr. Thomas H. Manley; Third Vice-President, Dr. E. F. Yancey; Fourth Vice-President, Dr. E. A. McGannon; Sixth Vice-President, Dr. E. G. Cochran; Seventh Vice-President, Dr. W. B. Blaksley; Secretary, J. M. Linnon; Assistant Secretary, Dr. J. H. Ford; Treasurer, Dr. S. Harvey Read. Executive Committee: A. J. Mullen, Jr., Dr. J. S. Murphy, A. A. Thompson, C. K. Cole. Galveston, Texas, was fixed upon as the next place of meeting, the date to be selected by the Executive Cemmittee.

---

THE American Electro-Therapeutic Association will hold its next annual meeting in Chicago, on September 12th, 13th and 14th.

